# Putative MicroRNA-mRNA Networks Upon Mdfi Overexpression in C2C12 Cell Differentiation and Muscle Fiber Type Transformation

**DOI:** 10.3389/fmolb.2021.675993

**Published:** 2021-10-19

**Authors:** Bo Huang, Yiren Jiao, Yifan Zhu, Zuocheng Ning, Zijian Ye, Qing X. Li, Chingyuan Hu, Chong Wang

**Affiliations:** ^1^ National Engineering Research Center for Breeding Swine Industry, Guangdong Provincial Key Lab of Agro-Animal Genomics and Molecular Breeding, Guangdong Laboratory for Lingnan Modern Agriculture, College of Animal Science, South China Agricultural University, Guangzhou, China; ^2^ Department of Molecular Biosciences and Bioengineering, University of Hawaii at Manoa, Honolulu, HI, United States; ^3^ Department of Human Nutrition, Food and Animal Sciences, University of Hawaii at Manoa, Honolulu, HI, United States

**Keywords:** Mdfi, C2C12 cell differentiation, muscle fiber type transformation, MiRNA-mRNA interaction network, hub microRNA, hub gene

## Abstract

Mdfi, an inhibitor of myogenic regulatory factors, is involved in myoblast myogenic development and muscle fiber type transformation. However, the regulatory network of Mdfi regulating myoblasts has not been revealed. In this study, we performed microRNAs (miRNAs)-seq on Mdfi overexpression (Mdfi-OE) and wild-type (WT) C2C12 cells to establish the regulatory networks. Comparative analyses of Mdfi-OE *vs*. WT identified 66 differentially expressed miRNAs (DEMs). Enrichment analysis of the target genes suggested that DEMs may be involved in myoblast differentiation and muscle fiber type transformation through MAPK, Wnt, PI3K-Akt, mTOR, and calcium signaling pathways. miRNA-mRNA interaction networks were suggested along with ten hub miRNAs and five hub genes. We also identified eight hub miRNAs and eleven hub genes in the networks of muscle fiber type transformation. Hub miRNAs mainly play key regulatory roles in muscle fiber type transformation through the PI3K-Akt, MAPK, cAMP, and calcium signaling pathways. Particularly, the three hub miRNAs (miR-335-3p, miR-494-3p, and miR-709) may be involved in both myogenic differentiation and muscle fiber type transformation. These hub miRNAs and genes might serve as candidate biomarkers for the treatment of muscle- and metabolic-related diseases.

## Introduction

Myogenic differentiation is an essential process in myogenesis, regulated by specific transcription factors such as myoblast determination protein (Myod), myogenin (Myog), myogenic factor 5 (Myf5), and myogenic regulatory factor 4 (Mrf4) (Luo et al., 2013). Loss of muscle mass and function due to aging, injury, and muscle diseases decreases quality of life (Argiles et al., 2016). Thus, it is of considerable significance to understand the muscle myogenic differentiation mechanism for treating human muscle-related diseases.

Muscle fiber is the basic unit of skeletal muscle. On the basis of myosin heavy chains (MyHC), muscle fiber can be classified into type I (MyHC I), type IIa (MyHC IIa), type IIx (MyHC IIx), and type IIb (MyHC IIb) (Schiaffino and Reggiani, 2011). Slow-twitch muscles mainly contain type I and type IIa muscle fibers, which have high hemoglobin and mitochondria content, and a strong resistance to fatigue resistance. Fast-twitch muscles mostly contain type IIb muscle fibers, displaying low hemoglobin and mitochondria content, and have a weak resistance to fatigue resistance (Chen X. et al., 2018). Human metabolic diseases, such as insulin resistance, type 2 diabetes, and hypertension, are usually accompanied with the transformation of muscle fiber types (Oberbach et al., 2006; Lu et al., 2017; Aguirre et al., 2020). Therefore, it is essential to elucidate the mechanism of muscle fiber type transformation in order to develop remedies for metabolic-related diseases.

microRNAs (miRNAs) are a class of endogenous, non-coding, short (21–25 nucleotides) RNAs that post-transcriptionally regulate protein-coding genes through interaction with the mRNAs 3′ -untranslated region (Jiao et al., 2018). Many miRNAs play vital roles in skeletal muscle development, including cell proliferation, differentiation, and apoptosis processes (Vienberg et al., 2017). Muscle-specific miRNAs (MyomiRs) are highly expressed in skeletal muscle, including miR-1, miR-133a, miR-133b, and miR-206 (Zhang et al., 2018). They primarily target myogenic transcription factors, such as MyoD (Wu et al., 2019) and serum response factor (SRF) (Kim et al., 2006), to regulate the proliferation and differentiation of skeletal muscle cells. Besides MyomiRs, there are many other miRNAs involved in muscle development regulation. For instance, miR-27a/b act on the myostatin (MSTN) gene to promote myogenic cell proliferation by inhibiting MSTN (McFarlane et al., 2014). miR-29a/b/c and miR-24 target the transcriptional activator SMAD family gene and up-regulate the myogenic differentiation process (Sun et al., 2008; Wei et al., 2013). Myogenic regulatory factor inhibitor (Mdfi) interacts with the MyoD family members. The regulation of its interaction on downstream miRNAs expression is, however, unknown. Therefore, it is of significance to know the relationship between Mdfi and miRNAs for the next step of research on the muscle development regulation mechanism.

Mdfi is a MyoD family inhibitor, also known as I-mfa, which was reported to play a negative role in regulating the MyoD family’s transcription in the myogenic differentiation of NIH-3T3 cells (Chen et al., 1996). Injection of lentivirus vectors containing Mdfi interfering fragments into leg muscles of a 3-week-old mouse showed that interference with Mdfi significantly promoted the hypertrophy of mouse leg muscle fibers (Hou et al., 2018). We previously constructed a Mdfi-overexpressing C2C12 cell line by the CRISPR/Cas9 system and found that overexpression of Mdfi promoted the C2C12 cell myogenic differentiation and muscle fiber transformation from type IIb to type IIa and type I (Huang et al., 2021).

In the present study, we proposed the miRNA-mRNA networks to further explore the function of Mdfi on C2C12 cell myogenic differentiation and muscle fiber type transformation. We selected differentiated C2C12 cells with Mdfi overexpression (Mdfi-OE) and wild-type (WT) for miRNA-seq analysis. We proposed pathways in which differentially expressed miRNAs (DEMs) perform essential functions in myogenic differentiation and muscle fiber type transformation. We also proposed a regulatory network of overexpression of Mdfi on C2C12 cell differentiation and muscle fiber type transformation and identified putative hub miRNAs and genes in them. These findings help to explain how Mdfi regulates myogenic development and muscle fiber type transformation. These hub miRNAs and genes might provide candidate biomarkers for treating muscle- and metabolic-related diseases.

## Materials and Methods

### Preparation of Cells and Cell Culture

In this study, we selected the mouse skeletal muscle cell line (C2C12), which was preserved in our laboratory (South China Agricultural University, Guangzhou, China) as the experimental cells. The C2C12 cells were cultivated in a basal medium containing Dulbecco’s modified Eagle’s medium (DMEM, Gibco, Grand Island, New York, NY, United States ) with 10% fetal bovine serum. C2C12 cells were seeded in 6-well plates (2 × 10^5^ cells per well). When reached 80–90% confluence, the cells were cultured by myogenic differentiation induction medium (with 2% horse serum) (Hou et al., 2019). We previously constructed an Mdfi stably overexpressing cell line from C2C12 cells by CRISPR/Cas9 (Huang et al., 2021). Our previous studies showed that overexpression of Mdfi promoted C2C12 cell differentiation and positively modulated muscle fiber type transformation (Huang et al., 2021). In the present study, we collected experimental samples (Mdfi-OE) from differentiated C2C12 cells on day 5 and used them for further miRNA-seq analysis.

### RNA Extraction and Sequencing

Total RNA was extracted from two groups of six C2C12 cell samples in triplicate with Trizol reagent (Invitrogen, Carlsbad, CA, United States ). The purity of RNA was checked with a NanoDrop ND-2000 spectrophotometer (Thermo Scientific, Waltham, CA, United States ). The integrity of total RNA was assessed with the RNA Nano 6000 assay kit of the Agilent Bioanalyzer 2100 system (Agilent Technologies, Santa Clara, CA, United States ) (Hou et al., 2017). After library preparation, entire libraries from six samples were sequenced according to both 50 bp single-end miRNA methods based on the Illumina Hiseq platform (Caporaso et al., 2012).

### Analysis of miRNA Sequencing Data

We used Trimmomatic software to remove the adapter and low-quality sequences from raw data and to clean reads. The sequences from 18 to 32 nt were considered to be high-quality clean reads. After quality control, clean reads were mapped to the mouse reference genome (*Mus musculus* 10.fa) using BWA software. By using blast, the mapped reads were aligned to the mirbase version 22 (www.mirbase.org) (Kozomara et al., 2019) to identify known miRNAs. The remaining reads were used to detect other small RNA in the Rfam databases (rfam.janelia.org) (Kalvari et al., 2018) by blast software. Differential expression analysis was performed on DESeq2 software, and read counts were the main data. miRNA-seq data was normalized by DESeq2 normalization (Love et al., 2014). The volcano plot and heatmap were generated by (https://software.broadinstitute.org/morpheus) to display an overview of the DEMs. The upset plots were generated by webtool (https://intervene.shinyapps.io/intervene/) (Khan and Mathelier, 2017). The *q* < 0.05 and |Log2FoldChange| > 1 was set as the cutoff criteria for differentially expressed analysis.

### Quantitative Real Time-PCR of the Differentially Expressed miRNAs

To confirm differential expression analysis results, we randomly selected DEMs for quantitative PCR verification. The internal reference gene was U6 in the DEM. The primer sequences designed for the DEMs are listed in Supplementary Table S1. The qRT-PCR reactions were as follows: 40 cycles of 95°C for 20 s, 60°C for 20 s, and 72°C for 20 s. All data were expressed as the mean ± standard error of the mean (S.E.M.). The assumptions of normality of data and homogeneity of variances between the groups were analyzed with SPSS. Significant differences between treatment groups were determined *via* one-way ANOVA (SPSS 18.0, Chicago, IL, United States ). A value of *p* < 0.05 was considered to be statistically significant. * stands for *p* < 0.05, while ** stands for *p* < 0.01.

### Gene Set Enrichment Analysis

Gene Ontology (GO) enrichment analyses of genes were conducted using the Database for Annotation, Visualization, and Integrated Discovery (DAVID, david.ncifcrf.gov) (Huang et al., 2009). Kyoto Encyclopedia of Genes and Genomes (KEGG) enrichment analyses of genes were conducted using KOBAS 3.0 (kobas.cbi.pku.edu.cn) (Xie et al., 2011). We used GO enrichment analysis to infer the biological function of DEGs. GO terms including three categories: biological process (BP), cellular component (CC), and molecular function (MF). The KEGG database analysis was used to analyze the enriched pathways for the DEGs. The Benjamini-Hochberg procedure was applied to control the false discovery rate (*p* < 0.05).

### Construction of miRNA–mRNA Interaction Network

We used two strategies to establish miRNA-mRNA regulatory networks: predictive target genes and negative regulatory relationships. Prediction of multiple DEM target genes was performed with the DIANA database (Karagkouni et al., 2018). According to the principle of miRNA’s negative regulatory gene, we overlapped the predicted DEM target genes and DEGs to establish miRNA-mRNA interaction networks. We then utilized Cytoscape 3.6 to analyze and visualize the miRNA-mRNA network.

## Results

### Overview of miRNA Sequencing Data

The miRNA profiles were generated by sequencing six C2C12 cell samples (three each from Mdfi-OE and WT). The sample’s length, clean reads, Q30 quality scores, GC content, and unique mapped rate were summarized in Supplementary Table S2. After filtration, a total of 63.49 million clean reads were retrieved from the miRNA profiles, of which more than 89% were mapped to the mouse reference genome. The length of the clean reads ranged from 22 to 25 nt, Q30 quality scores of the six samples were above 97%, and the samples’ GC content ranged from 46 to 49%. These data met the standard analysis requirements and could be further analyzed.

### Differentially Expressed miRNA Analysis in Mdfi-OE Versus WT

**FIGURE 1:**
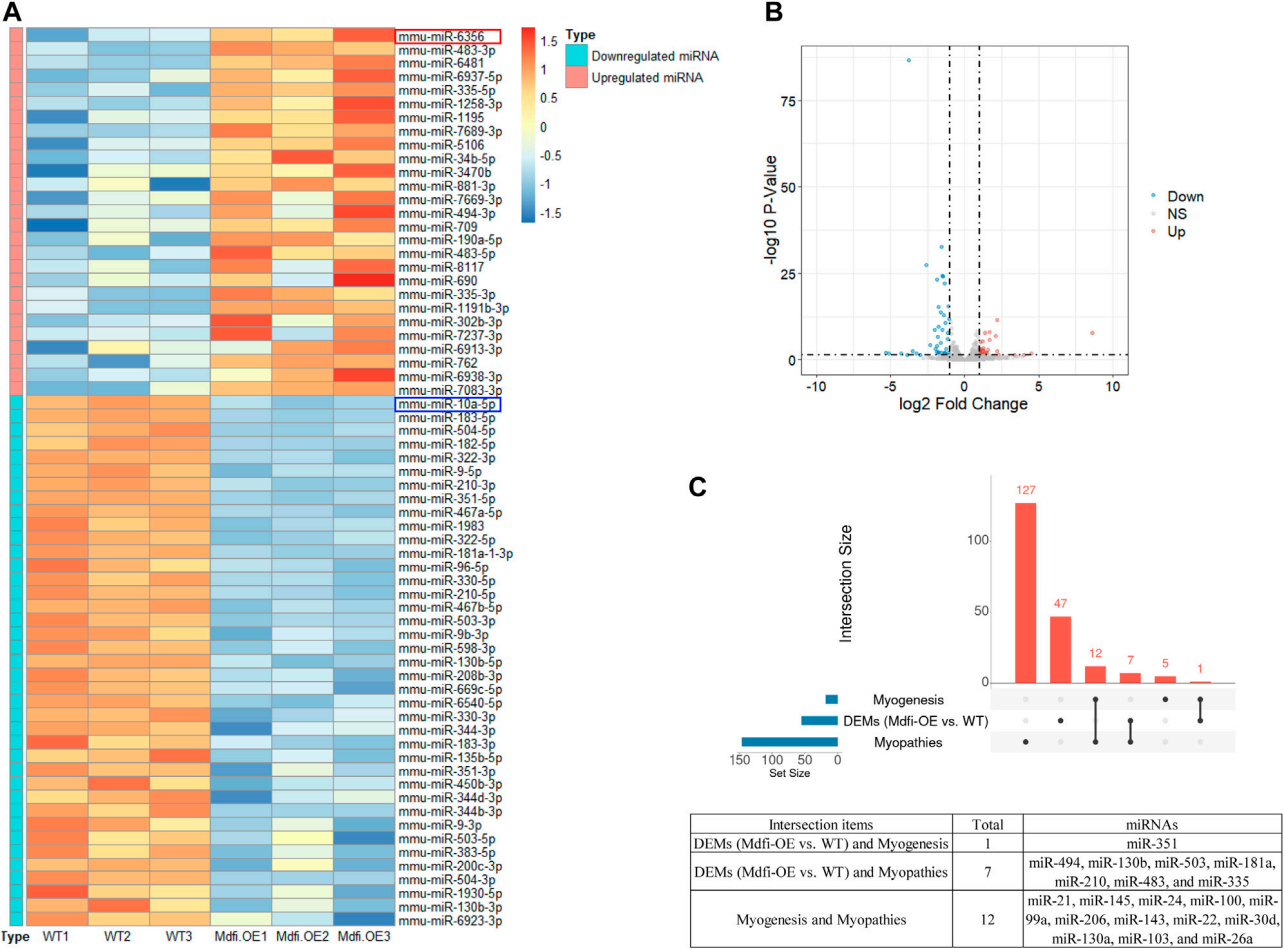
The overview of the DEMs. (**A)**. The Heatmap plot. Samples: Wild-Type 1/2/3 represent normal C2C12 cells. Mdfi-Overexpression 1/2/3 represent overexpression Mdfi in C2C12 cells. The types include upregulated miRNAs and downregulated miRNAs. Normalized log_2_ data were hierarchically clustered by miRNA and are plotted as a heatmap. A color scale represents the relative intensity of the expression of miRNA. Orange represents high expression and blue represents low expression. **(B)**. The Volcano plot. The X-axis represents log-transformed *p*-value, and the Y-axis indicates the multiple of the DEMs between Mdfi-OE and WT groups. Note: the grey dots represent DEMs that are not differentially expressed, the red dots represent the upregulated DEMs, and the blue dots represent the downregulated DEMs. |Log2FoldChange| > 1 and *p*-value < 0.05 were set as the criteria. Down, NS, and Up represent the downregulated, not significant, and upregulated DEMs. **(C)**. The intersection analysis plot. UpSet plot of the intersection analysis of DEM, myogenesis and myopathie. The transverse bar graph at the bottom left shows the number of miRNAs. The bars above represent the number of miRNAs corresponding to each intersection.

We performed miRNA-Seq to identify microRNAs (miRNAs) differentially expressed between Mdfi-OE and WT groups in triplicate per group. Based on the criteria of *q* < 0.05 and |Log2FoldChange| > 1, we identified 66 differentially expressed miRNAs (DEMs), among which 27 miRNAs were upregulated, and 39 miRNAs were downregulated by overexpression of Mdfi in C2C12 cells (Supplementary Table S3). The Heatmap and Volcano plot were generated to display an overview of the DEMs (Figures 1A,B). The most abundant upregulated DEM was miR-6356, whose function has not been reported before. The most significant difference downregulated DEM was miR-10a-5p, which belonged to the miR-10 family. The function of miR-10a-5p in skeletal muscle differentiation has not been reported before. However, miRNA-10b, another member of the miRNA-10 family, targeted the nuclear factor of activated T-cells (NFAT) to inhibit myoblast differentiation (Ge et al., 2019). Therefore, the function of miRNA-10 in myogenic differentiation warrants further investigation. The top 10 upregulated and downregulated DEMs with a significant difference (*p* < 0.05) were listed in Table 1. We also identified four miRNAs (miR-34b-3p, miR-494-3p, miR-182-5p, and miR-351-5p) that have been reported to involve in muscle development and muscle fiber type transformation.

**TABLE 1:** Top 10 DEMs with a significant difference.

Upregulated DEM			Downregulated DEM		
miRNA	log_2_ (FC)	*p* value	miRNA	log_2_ (FC)	*p* value
mmu-miR-6356	2.20	3.45E-12	mmu-miR-10a-5p	−3.75	3.18E-87
mmu-miR-483-3p	1.69	9.08E-09	mmu-miR-183-5p	−1.56	2.84E-33
mmu-miR-6481	8.61	2.08E-08	mmu-miR-504-5p	−2.57	3.40E-28
mmu-miR-6937-5p	1.39	2.08E-08	mmu-miR-182-5p	−1.50	5.12E-25
mmu-miR-335-5p	2.11	1.14E-07	mmu-miR-322-3p	−1.47	8.66E-25
mmu-miR-1258-3p	1.70	1.77E-06	mmu-miR-9-5p	−1.89	7.85E-24
mmu-miR-1195	1.23	3.92E-06	mmu-miR-210-3p	−1.37	8.20E-23
mmu-miR-7689-3p	1.07	8.32E-06	mmu-miR-351-5p	−1.09	3.51E-16
mmu-miR-5106	1.24	0.000467	mmu-miR-467a-5p	−1.74	4.25E-16
mmu-miR-34b-5p	1.58	0.001123	mmu-miR-1983	−1.60	1.53E-14

### Intersection Analysis of DEM, Myogenesis and Myopathies

miRNA is not only involved in the regulation of muscle development (Mok et al., 2017), but also related to the occurrence of muscle diseases (Sayed and Abdellatif, 2011). Therefore, we analyzed the intersection of DEMs, miRNAs involved in myogenesis (Chen et al., 2006), and miRNAs involved in myopathies (Eisenberg et al., 2007). As shown in the Figure 1C, miR-351-5p is muscle-specific miRNA identified in DEMs; In the DEMs, seven miRNAs were associated with muscle disease, such as miR-494-3p, miR-130b, and miR-335-5p. In particular, miR-335 was found upregulated in seven muscle disorders and was the same direction of expression when Mdfi was overexpressed. miR-335 is also the hub miRNA in the miRNA-mRNA interaction network. These hub miRNAs may be the candidate biomarkers for treating muscle-related diseases.

### Quantitative Real-Time PCR Validation

**FIGURE 2:**
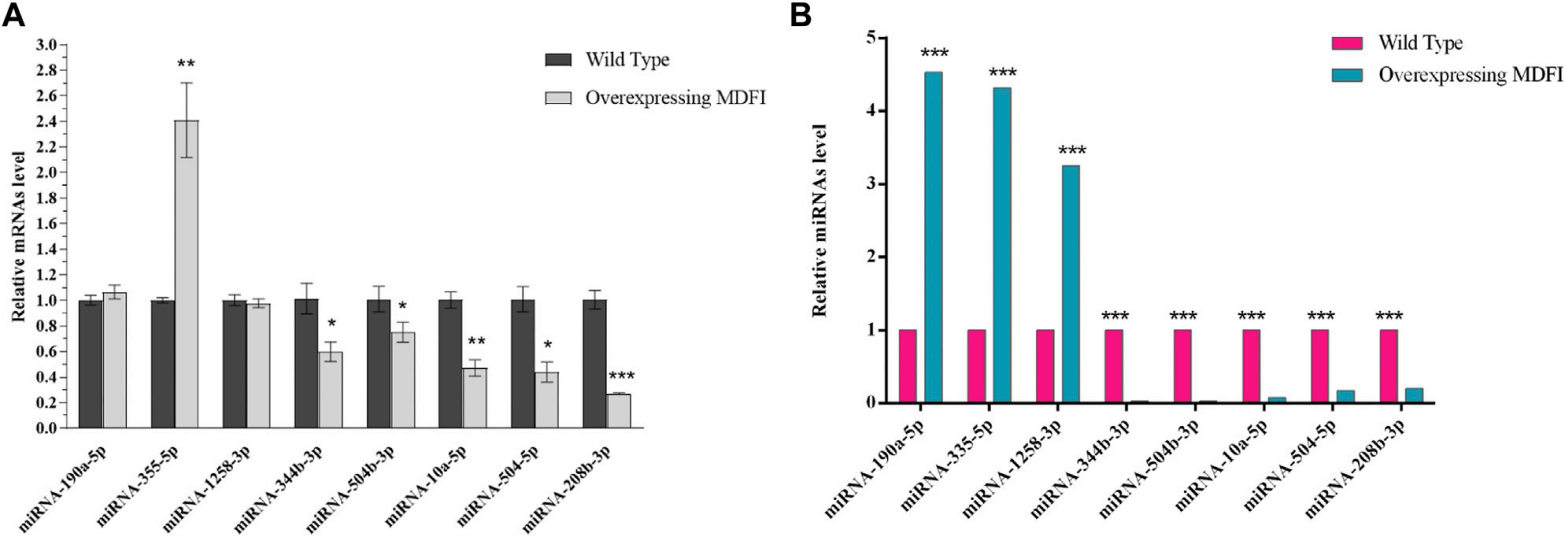
Quantitative RT-PCR validation. **(A)**. qRT-PCR validated of DEMs. **(B)**. Expression levels of DEMs in miRNA-seq. **p < 0.01, ***p < 0.001.

To validate the DEMs identified by RNA-seq data, we randomly selected eight DEMs, including two hub miRNAs (miR-335-5p and miR-10a-5p), for analysis by qPCR in Mdfi-OE *vs*. WT. The qPCR results for DEMs were consistent with the results of the miRNA-seq data, except for miR-190-5p and miR-1258-3p (Figure 2). Thus, the qPCR results had a high consistency rate (75%) with miRNA-seq results, which indicates good reliability of our sequencing data.

### Prediction and Functional Analysis of Target Genes of DEMs

We herein used DIANA Tools (version 2.0) to predict the target genes of each DEMs (Supplementary Table S4). There were 2074 target genes for 27 upregulated DEMs, consisting of 2454 miRNA-mRNA interaction pairs. We also predicted 2,846 target genes for 39 downregulated DEMs, consisting of 4,083 miRNA-mRNA interaction pairs.

**FIGURE 3:**
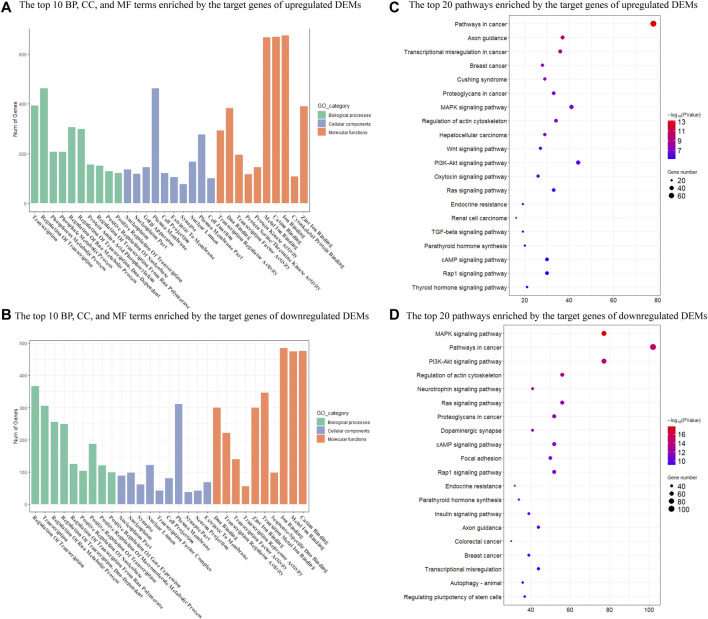
Enrichment analysis of target genes of DEMs in Mdfi-OE vs. WT. **(A)**. The first 10 biological processes (BP), 10 cellular component (CC), and 10 molecular function (MF) are enriched by the target genes of upregulated DEMs. **(B)**. The top ten biological processes (BP), cellular component (CC), and molecular function (MF) are enriched by the target genes of downregulated DEMs. **(C)**. The first twenty pathways of the target genes of upregulated DEMs. **(D)**. The first twenty pathways of the target genes of downregulated DEMs.

We then performed GO and KEGG enrichment analysis on target genes. GO functional enrichment analyses revealed that 755 and 1189 GO terms were significantly enriched by upregulated and downregulated target genes (*p* < 0.05), respectively (Supplementary Tables S5, S6). These GO terms were mainly involved in the regulation of transcription, transcription regulator, and nuclear. The top 10 GO terms in each category with a significant difference were shown in Figure 3. There were also 145 KEGG pathways enriched by the target genes of upregulated DEMs (*p* < 0.05), including the Pathways in cancer, Axon guidance, MAPK signaling pathway, and Regulation of actin cytoskeleton (Supplementary Table S7). There were a total of 184 KEGG pathways enriched by the target genes of downregulated DEMs (*p* < 0.05), including the pathways in cancer, MAPK signaling pathway, regulation of actin cytoskeleton, and PI3K-Akt signaling pathway (Supplementary Table S7). The top 20 significantly enriched pathways were shown in Figure 3.

### Integrated Analysis of DEMs and Differentially Expressed Genes in C2C12 Cell With Overexpressed Mdfi

**FIGURE 4:**
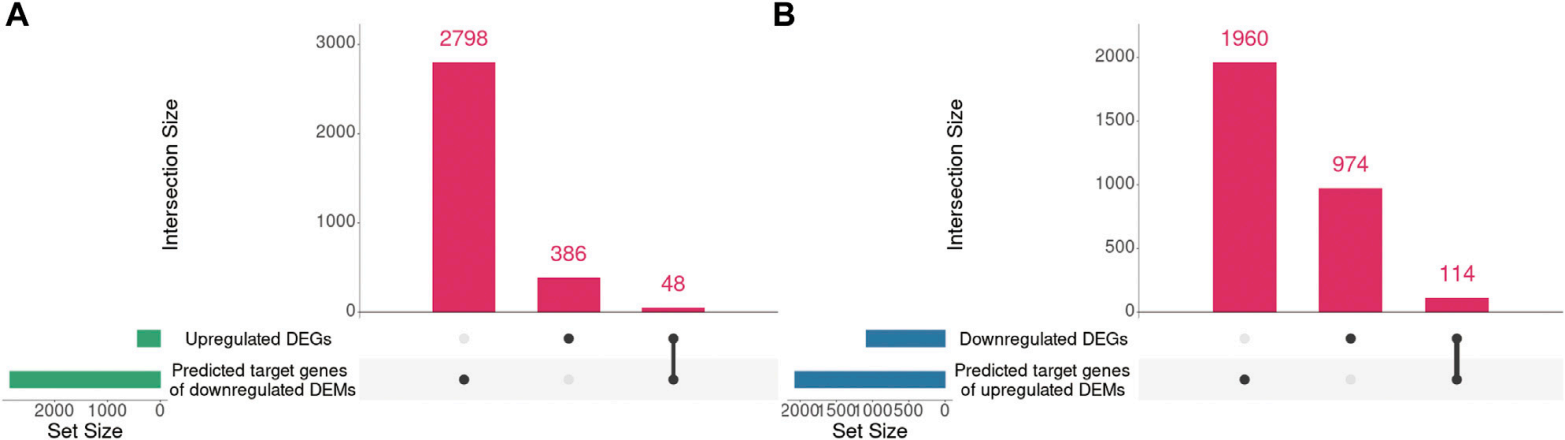
The intersection of DEGs and predicted target genes of DEMs. **(A)**. The intersection of upregulated DEGs and predicted target genes of downregulated DEMs. **(B)**. The intersection of downregulated DEGs and predicted target genes of upregulated DEMs. UpSet plot of the intersection of DEGs and predicted target genes of DEMs. The transverse bar graph at the bottom left shows the number of genes enriched in each pathway. The black dots at the bottom left and the bar graph at the top indicate genes. The bars above represent the number of genes corresponding to each intersection.

Our previous mRNA-seq analysis identified 434 upregulated DEGs and 1088 downregulated DEGs in Mdfi-OE *vs*. WT. Mdfi was upregulated 64-fold in the mRNA-seq results (Huang et al., 2021). In the present study, we intersected the prediction target genes of DEMs with these DEGs and formed miRNA-mRNA interaction pairs based on miRNA’s negative regulation genes. A total of 48 genes were overlapped between the 434 upregulated DEGs and 2,846 predicted target genes of downregulated DEMs, and a total of 114 genes were overlapped between the 1,088 downregulated DEGs and 2,074 predicted target genes of upregulated DEMs (Figure 4).

**FIGURE 5:**
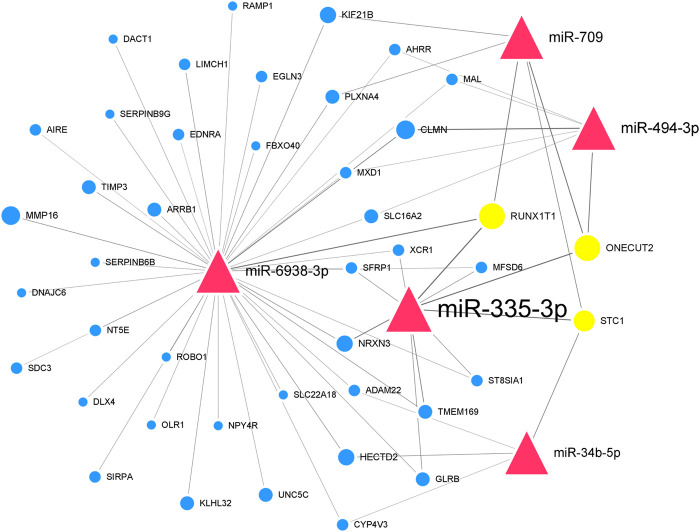
Thu hub upregulated miRNA-downregulated mRNA interaction network in Mdfi-OE vs. WT.The network displays the hub miRNA-mRNA interactions. Nodes represent miRNA and mRNA, and edges represent interactions. The upregulated miRNAs are shown in red, downregulated genes are shown in blue, and hub genes are shown in gold. The miRNAs are depicted by triangles and the genes as circles.

We further paired 23 upregulated miRNAs with the above 114 downregulated genes and 25 downregulated miRNAs with the above 48 upregulated genes to form 122 and 71 miRNA-mRNA interactions, respectively. After integrated analysis, we used Cytoscape 3.6 software to display miRNA-mRNA interaction networks. The top 5 significant upregulated miRNAs in the upregulated miRNA-downregulated mRNA interaction network were miR-335-3p (degree = 120), miR-494-3p (degree = 45), miR-6938-3p (degree = 41), miR-709 (degree = 41), and miR-34b-5p (degree = 31) (Supplementary Figure S1). Many downregulated genes were also regulated by multiple miRNAs. Runx1t1 (degree = 8), Onecut2 (degree = 8), and Stc1 (degree = 6) were targeted by three hub miRNAs (Table 2). We further selected the first neighbors of these five hub miRNAs to construct the hub upregulated miRNA-downregulated mRNA interaction network (Figure 5).

**TABLE 2:** The hub miRNAs and genes.

Upregulated miRNAs		Downregulated miRNAs	
miRNAs	Target DEGs	miRNAs	Target DEGs
miR-335-3p	R unx1t1, Onecut2, Stc1	miR-330-5p	Ttn, Slc5a3
miR-494-3p	ONECUT2 necut2	miR-9-5p	Ttn, Slc5a3
miR-6938-3p	R unx1t1	miR-322-5p	Ttn, Slc5a3
miR-709	R unx1t1, ONECUT2 necut2, Stc1	miR-96-5p	Ttn, Slc5a3
miR-34b-5p	Stc1	miR-182-5p	Ttn, Slc5a3

**FIGURE 6:**
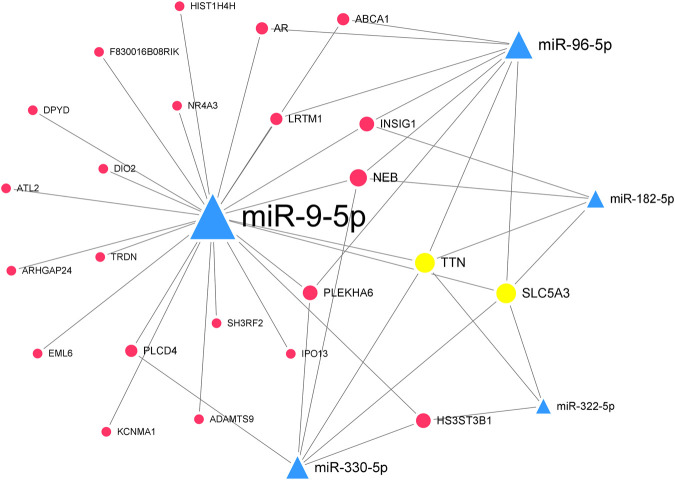
The hub downregulated miRNA-upregulated mRNA interaction network in Mdfi-OE vs. WT.The network displays the hub miRNA-mRNA interactions. Nodes represent miRNA and mRNA, and edges represent interactions. The downregulated miRNAs are shown in green, upregulated genes are shown in blue, and hub genes are shown in gold. The miRNAs are depicted by triangles and the genes as circles.

In the downregulated miRNA-upregulated mRNA interaction network, the top 5 significant downregulated miRNAs were miR-330-5p (degree = 24), miR-9-5p (degree = 23), miR-322-5p (degree = 22), miR-96-5p (degree = 19), and miR-182-5p (degree = 18) (Supplementary Figure S2). The upregulated genes, Ttn (degree = 13) and Slc5a3 (degree = 9), were targeted by all five hub miRNAs (Table 2). Similarly, we constructed the hub downregulated miRNA-upregulated mRNA interaction network (Figure 6).

### Integrated Analysis of DEMs and DEGs of Overexpressed Mdfi on Muscle Fiber Type Transformation

Overexpression of Mdfi positively modulates fast-to-slow-twitch muscle fibers transformation (Huang et al., 2021). We herein used miRNA-seq analysis to identify the miRNAs involved in muscle fiber type transformation. We selected the 101 DEGs related to the transformation of muscle fiber types in our previous study and matched these DEGs with the DEMs in the miRNA-seq.

**FIGURE 7:**
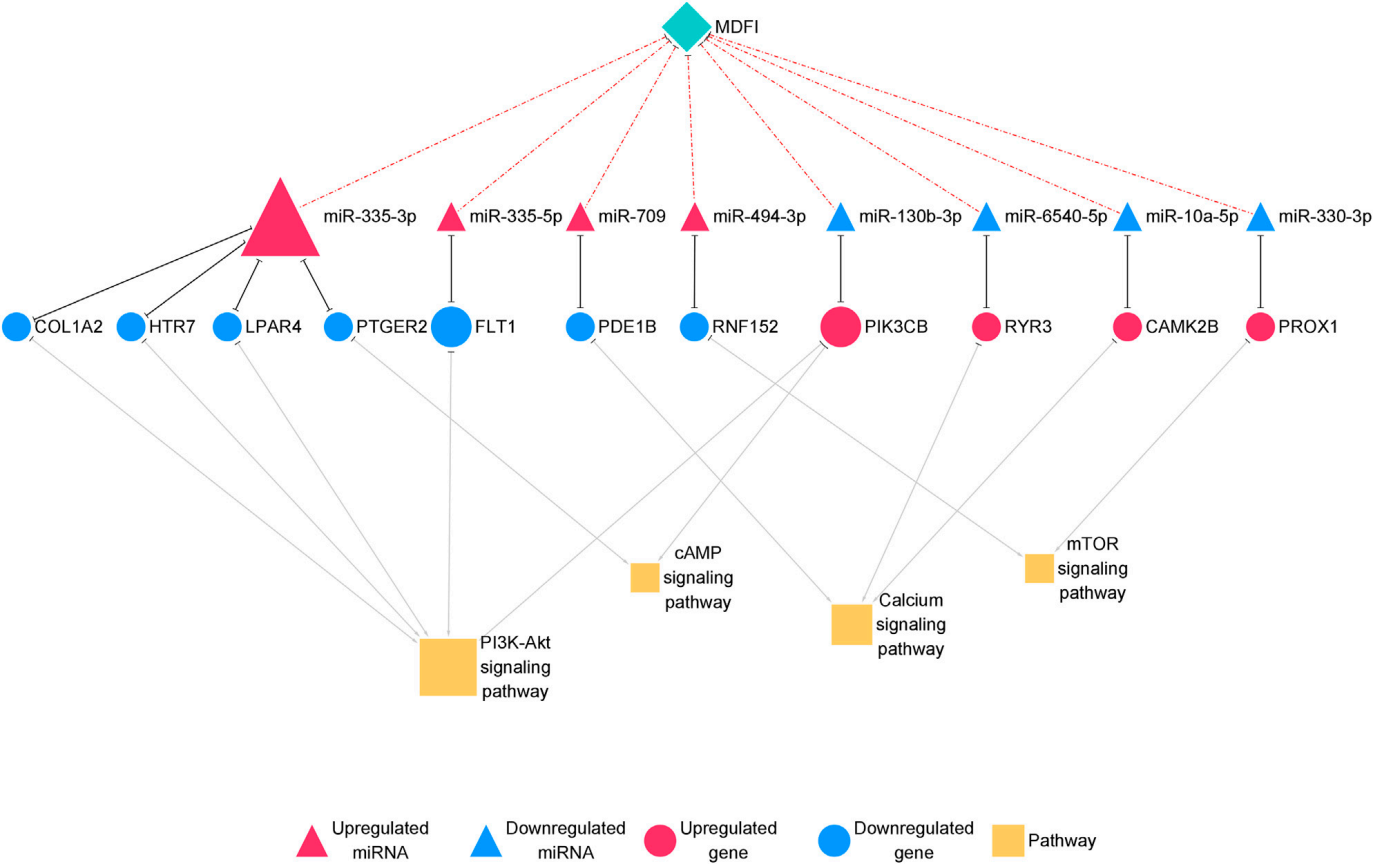
The miRNA-mRNA interaction network related to muscle fiber type transformation.The network displays miRNA-mRNA interactions. Nodes represent miRNA and mRNA, and edges represent gene interactions. The miRNAs are shown in red; genes are shown in blue, and pathways are shown in yellow. The miRNAs are depicted by triangles, the mRNAs as circles, and the pathways as squares.

Furthermore, we proposed miRNA-mRNA interaction networks related to muscle fiber type transformation (Figure 7) and identified 11 miRNA-mRNA interaction pairs. These interaction pairs included four upregulated miRNAs, four downregulated miRNAs, four upregulated genes, and seven downregulated genes. In the upregulated miRNAs, miR-335-3p, miR-494-3p, and miR-709 were also hub miRNAs in the miRNA-mRNA interaction network of Mdfi overexpressed differentiated C2C12 cells. This result indicates that these miRNAs may be involved not only in the process of myogenic differentiation but also in muscle fiber type transformation.

## Discussion

We previously constructed an Mdfi-overexpressing C2C12 cell line, performed mRNA-seq on Mdfi-OE and WT C2C12 cells. Overexpression of Mdfi promoted C2C12 cell differentiation and positively modulated muscle fiber type transformation (Huang et al., 2021). Mdfi overexpression may not be the only possibility to regulate myoblasts differentiation. Many factors may regulate myoblasts differentiation, but the regulatory pathways are different. For example, MYOC regulates TGFb by inhibiting CAV1 to promote myoblasts differentiation. Lmod3 promotes differentiation of C2C12 cells by activating the AKT and ERK pathways. However, the regulatory network in Mdfi-overexpressing C2C12 cells has not been revealed. In this study, we performed miRNA-seq on Mdfi-OE and WT C2C12 cells. We drafted miRNA-mRNA interaction networks involved in myogenic differentiation and muscle fiber type transformation regulated by Mdfi, and identified hub miRNAs and genes in networks. These results may help in understanding the mechanisms of Mdfi in myogenic development and muscle fiber type transformation.

### Differentially Expressed miRNA Analysis in Mdfi-OE Versus WT

In the comparative analysis of Mdfi-OE *vs*. WT, we identified 66 DEMs. Among these DEMs, four miRNAs (miR-34b-3p, miR-494-3p, miR-182-5p, and miR-351-5p) have been reported to involve in muscle development and muscle fiber type transformation. miR-34b-3p promoted the myogenic differentiation of C2C12 cells and produced more multicore myotubes (Tang et al., 2017). miR-351-5p targeted lactamase beta (Lactb) to inhibit C2C12 cell differentiation and led to muscle fiber type transformation (Du et al., 2019). The knockout of miR-182-5p promoted muscle fibers’ transformation from type IIa to type I (Zhang et al., 2016). The overexpression of miR-494-3p inhibited myotube formation and the expression of MYH2 during human skeletal myogenesis (Yamamoyo et al., 2012). The expression trend of three miRNAs (miR-34b-3p, miR-351-5p, and miR-182-5p) was consistent with our previous results that overexpression of Mdfi promotes the differentiation of C2C12 cells and muscle fiber type transformation. However, based on the function of miRNA-494 previously reported, the expression trend of miR-494-3p in miRNA-seq was inconsistent with our previous results. We hypothesized that a novel interaction might exist between Mdfi and miR-494-3p.

### Functional Analysis of Target Genes of DEMs in Mdfi-OE Versus WT

The enriched GO terms of upregulated and downregulated DEMs target genes were mainly involved in the regulation of transcription, nuclear, and metal ion binding and transport. Mdfi plays essential roles in inhibiting protein transport into the nucleus and ultimately inhibiting its transcriptional activity (Chen et al., 1996). These results suggested that DEM may regulate gene entry into the nucleus and its transcriptional activity in C2C12 cells with overexpressed Mdfi.

The KEGG pathways enriched by upregulated and downregulated DEMs target genes, especially MAPK signaling pathway (Troy et al., 2012), Wnt signaling pathway (Girardi and Le Grand, 2018), PI3K-Akt signaling pathway (Rommel et al., 2001), mTOR signaling pathway (Go et al., 2020), and calcium signaling pathway (Swoap et al., 2000), and are all known to be involved in myogenic differentiation and muscle fiber type transformation. In particular, PI3K/AKT signaling pathway activation has been reported to induce myomiR maturation in C2C12 cells (Briata et al., 2012). mTOR is a crucial pathway signaling pathway of skeletal muscle hypertrophy associated with increased protein synthesis (Go et al., 2020). mTOR regulates mitochondrial function and participate in glycolytic flux and mitochondrial respiration (Tan et al., 2015). The increase of intracellular cAMP level increases the expression of CaMKIV and PGC-1 in myoblast cells, and then regulate the formation of muscle fibers (Handschin et al., 2003). There is feedback regulation between cAMP and intracellular calcium homeostasis, which plays an important role in the regulation of muscle fiber transformation(Walke et al., 1994). miRNA-21 has been reported to inhibit PI3K/Akt/mTOR signaling by targeting TGFBI during skeletal muscle development (Briata et al., 2012). Muscle development is associated with calcium homeostasis, and many miRNAs in calcium signaling appeared to be suppressed in aged skeletal muscles (Yan et al., 2018). These results suggest that DEM may regulate myoblast differentiation and transform muscle fiber types through these signaling pathways.

### Identification of Hub miRNAs and Genes in Regulatory Networks Associated With Mdfi Overexpression on C2C12 Cells Differentiation

To investigate how miRNA regulates the gene expression of overexpressed Mdfi, we drafted miRNA-mRNA interaction networks associated with Mdfi overexpression on C2C12 cell differentiation. Among the networks, high degreed-nodes in the regulatory network are also known as hub miRNAs or genes.

In the hub upregulated miRNA-downregulated mRNA interaction network, we identified five hub miRNAs (miR-335-3p, miR-494-3p, miR-6938-3p, miR-709, and miR-34b-5p) and three hub genes [RUNX1 partner transcriptional co-repressor 1 (Runx1t1), one cut homeobox 2 (Onecut2), and stanniocalcin 1 (Stc1)]. These hub miRNAs paired with hub genes to form nine interaction pairs (miR-335-3p-Runx1t1/Onecut2/Stc1, miR-709-RUNX1T1unx1t1/Onecut2/STC1, miR-494-3p/ONECUT2necut2, miR-6938-3p/RUNX1T1unx1t1, and miR-34b-5p/Stc1). MiR-335-3p was induced during the differentiation of C2C12 cells and highly expressed during muscle regeneration (Greco et al., 2009). MiR-709 has been documented to be regulated adipocyte differentiation by targeting glycogen synthase kinase3b (Gsk3b) (Chen et al., 2014), but its function on muscle development has not been reported. MiR-494-3p has been reported to inhibit the differentiation of myoblast and lead to muscle fiber type transformation (Iwasaki et al., 2015). Still, its expression trend was inconsistent with the results of Mdfi promotes C2C12 cell differentiation. Therefore, we hypothesized that a novel interaction might exist between Mdfi and miR-494-3p, thereby regulating myoblast differentiation. To our knowledge, there are no published reports on miR-6938-3p. MiR-34b-5p was previously reported to promote the differentiation of C2C12 cells, and its upregulation is required for myotube formation (Tang et al., 2017). In our study, miR-34b-5p was upregulated, indicating that Mdfi may promote the differentiation of C2C12 cells by upregulating the expression of miR-34b-5p. Runx1t1 inhibited brown adipogenesis and inhibited white adipogenesis in 3T3 cells (Sun et al., 2011). Stc1 was involved in regulating calcium homeostasis in mammals and expressed from myoblast differentiation to myotube fusion. (Jiang et al., 2000). Onecut2 has been discovered as a transcriptional factor and regulated the hepatocyte differentiation (Francius and Clotman, 2010), but its function on muscle development has not been reported. In addition, in our enrichment analysis, these three hub genes were not enriched in any pathways involved in myogenic differentiation. All the above hub miRNAs and genes were significantly changed in our RNA-Seq results. They play essential roles in the process of Mdfi regulating C2C12 cell differentiation.

By contrast, we identified five hub miRNAs (miR-330-5p, miR-9-5p, miR-322-5p, miR-96-5p, and miR-182-5p) and two hub genes [titin (Ttn) and solute carrier family 5 member 3 (Slc5a3)] in the hub downregulated miRNA-upregulated mRNA interaction network. They formed ten interactions pairs (miR-330-5p/miR-9-5p/miR-322-5p/miR-96-5p/miR-182-5p-Ttn/Slc5a3). In the present study, miR-330-5p was proposed to bind STAT3, inhibiting muscle wasting (Mubaid et al., 2019). The overexpression of miR-9-5p significantly inhibited myoblasts differentiation, whereas the inhibition of miR-9-5p had the opposite effect (Yin et al., 2020). In our study, miR-9-5p was downregulated, indicating that Mdfi may promote the differentiation of C2C12 cells by downregulating miR-9-5p. The inhibition of miR-322-5p attenuated Dexamethasone (Dex)-induced muscle atrophy in C2C12 myotubes (Geng et al., 2020). MiR-96-5p has been reported to regulate proliferation, migration, and apoptosis of vascular smooth muscle cells (Tian et al., 2020), but its function on muscle cell differentiation remains unclear. MiR-182-5p was highly expressed in the mouse’s fast-twitch muscles, and knockout of miR-182-5p promoted MyHC I expression and reduced MyHC IIa expression (Zhang et al., 2016). TTN, an abundant filamentous protein, is activated by calcium. TTN promoted muscle development by increasing the expression of MYH6, myosin light chain 1 (MYL1) (Kelly et al., 2020). SLC5A3, also known as SMIT1, is expressed in cardiomyocytes, and its primary function is to detect increased extracellular glucose concentrations (Van Steenbergen et al., 2017).

All the above hub miRNA genes were significantly changed in the miRNA-Seq results. Therefore, we hypothesized that overexpression of Mdfi would promote C2C12 cell differentiation through these 19 interaction pairs, and the role of ten hub miRNAs and five genes contained in interaction pairs needs to be further explored. In particular, the hub miRNAs mentioned above, we identified two miRNAs (miR-182-5p and miR-494-3p), suggesting that overexpression of Mdfi may induce muscle fiber type transformation. Their functions need to be further explored.

### Integrated Analysis of Muscle Fiber Type Transformation in Response to Mdfi Overexpression

In the miRNA-mRNA interaction networks, we identified four hub miRNA (miR-335-3p, miR-335-3p, miR-494-3p, and miR-709) and seven hub genes [collagen type I alpha 2 chain (Col1a2), 5-hydroxytryptamine receptor 7 (Htr7), lysophosphatidic acid receptor 4 (Lpar4), prostaglandin E receptor 2 (Ptger2), fms related receptor tyrosine kinase 1 (Flt1), phosphodiesterase 1B (Pde1b), and ring finger protein 152 (Rnf152)]. These hub miRNAs paired with hub genes to form seven interaction pairs (miR-335-3p-Col1a2/Htr7/Lpar4/Ptger2, miR-335-5p-Flt1, miR-709-Pde1b, miR-494-3p-Rnf152). MiR-335-3p was previously reported to involve in the differentiation of myoblasts (Greco et al., 2009), but its function on muscle fiber type transformation has remained unclear. MiR-335-5p was shown to be involved in cardiac differentiation by regulating WNT and TGFb signaling pathways (Kay et al., 2019). To our knowledge, there are no published reports on the function of miR-709 in muscle fiber type transformation. As we described above, miR-494-3p has been reported to inhibit the expression of Myh2, the slow-twitch muscle fiber marker gene during human skeletal myogenesis (Iwasaki et al., 2015). However, Myh2 was upregulated in our previous results (Huang et al., 2021) and miR-494-3p was upregulated in this study, which was inconsistent with the literature reports. The expression trend of miR-494-3p was also inconsistent with our results of Mdfi positively modulates fast-to-slow-twitch muscle fibers transformation. Therefore, we hypothesized that it might be attributed to different species, or that a novel interaction may exist between Mdfi and miR-494-3p, thereby regulating the transformation of muscle fiber types. Col1a2 is a subtype of collagen 1. Collagen type I was proposed to markedly inhibit C2C12 cell differentiation and muscle regeneration, and increase collagen production (Alexakis et al., 2007). Htr7 promoted axon growth and synapse formation of the nerve (Olusakin et al., 2020), but its functions on muscle have not been reported before. Lpar4, also known as GPR23, is a G-protein coupled receptor and has been reported to increase intracellular concentrations of cAMP and calcium (Lee et al., 2007). Flt1 was downregulated in the myogenic differentiation and expressed in regenerating muscle fibers (Germani et al., 2003). Similarly, Flt1 was downregulated and identified as a hub gene in the miRNA-mRNA network of this study, suggesting possible regulation via miR-335-5p and involvement in muscle fiber type transformation. Pde1b is distributed in the human myocardium and activated by calcium/calmodulin (Wennogle et al., 2017). Rnf152 is an essential negative regulator of the mTOR pathway by targeting RagA (Deng et al., 2015). Rnf152 was downregulated and targeted by miR-494-3p in this network. Thus, Mdfi may downregulate Rnf152 via upregulating miR-494-3p, thereby regulating muscle fiber type transformation through the mTOR signaling pathway.

Similarly, we identified four hub miRNAs (miR-130-3p, miR-6540-5p, miR-10a-5p, and miR-330-3p) and four hub genes [phosphatidylinositol-4,5-bisphosphate 3-kinase catalytic subunit beta (Pik3cb), ryanodine receptor 3 (Ryr3), calmodulin-dependent protein kinase II beta (Camk2b), and Prospero homeobox 1 (Prox1)] in the downregulated miRNA-upregulated mRNA interaction network. They formed four interactions pairs (miR-130-3p-Pik3cb, miR-6540-5p-Ryr3, miR-10a-5p-Camk2b, miR-330-3p-Prox1). MiR-130-3p is closely related to the occurrence and development of cancer (Wang et al., 2020). There are no published reports on miR-6540-5p. The function of miR-10a-5p in muscle fiber type transformation has not been reported, but miRNA-10 family member, miRNA-10b, targeted NFAT to inhibit myoblast differentiation (Ge et al., 2019). Therefore, miRNA- 10a-5p may also be related to muscle development. Overexpression of miR-330-3p induced cardiac hypertrophy in mice (Chen Y. et al., 2018). In C2C12 cells, the knockout of Pik3cb delayed differentiation, whereas overexpression of Pik3cb promoted differentiation (Matheny et al., 2015). In skeletal muscle, Ryr3 is a sarcoplasmic reticulum calcium release channel (Protasi et al., 2000), which regulated the release and balance of calcium. Ryr3 was increased in our mRNA-seq results and enriched in the calcium signaling pathway, suggesting that Mdfi may regulate muscle fiber type transformation by regulating calcium concentration. Camk2b, a calcium-dependent kinase, promoted mitochondrial biogenesis, and participated in the formation of slow-twitch muscle fibers (Al-Shanti and Stewart, 2009). The knockout of Prox1 in the skeletal muscle caused slow to fast skeletal muscle fiber transformation (Petchey et al., 2014). In this study, Prox1 was upregulated; therefore, we thus speculated that Prox1 upregulation was involved in Mdfi regulated fast-to-slow- twitch muscle fibers transformation. These hub genes also were enriched in the PI3K-Akt signaling pathway, the cAMP signaling pathway, the calcium signaling pathway, and the mTOR signaling pathway. This result suggests that these hub miRNAs mainly play key regulatory roles in muscle fiber type transformation through these signaling pathways.

All the eight hub miRNAs and eleven genes in the interaction pairs mentioned above play essential roles in the process of Mdfi regulating the transformation of muscle fiber types. Their role needs to be further explored. In addition, three hub miRNAs (miR-335-3p, miR-494-3p, and miR-709) identified in the network of C2C12 cell differentiation were also identified as the hubs genes in the network of muscle fiber type transformation. Their functions need to be further explored.

## Conclusion

**FIGURE 8:**
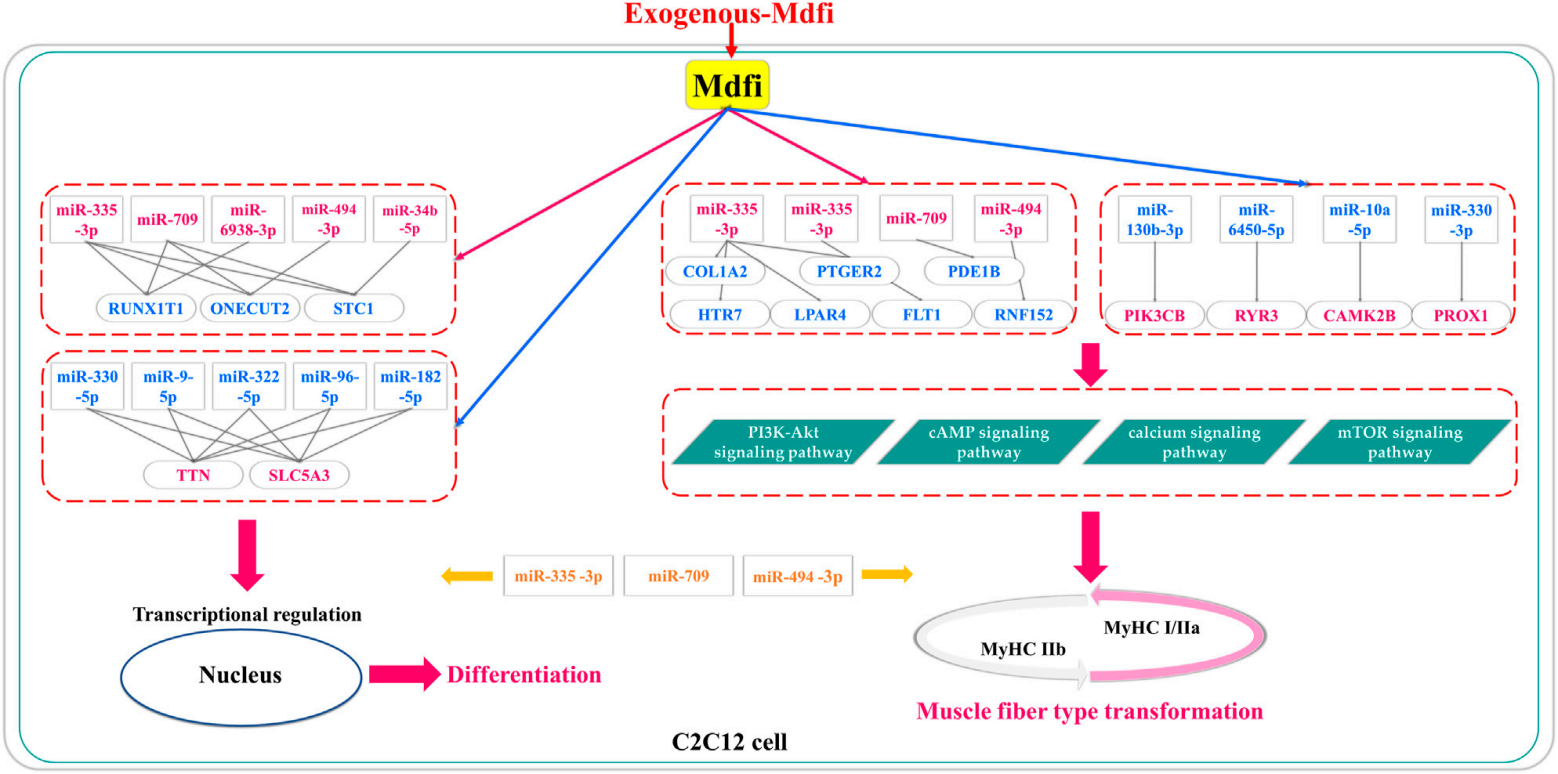
The putative miRNA-mRNA interaction networks for Mdfi overexpression on C2C12 cell differentiation and muscle fiber type transformation. The upregulations are shown in red, and the blue color represents the downregulation. In particular, miR-335-3p, miR-709, and miR-494-3p were involved in regulating both myogenic differentiation and muscle fiber type transformation.

In conclusion, based on miRNAs’ expression profiles, we performed differential analysis, enrichment analysis, and proposed miRNA-mRNA interaction networks of Mdfi overexpression on C2C12 cell differentiation and muscle fiber type transformation (Figure 8). We have putatively identified the pathways in which DEM performs its functions in myogenic differentiation and muscle fiber type transformation. Furthermore, we putatively identified hub miRNAs and genes in the miRNA-mRNA networks. In particular, three hub miRNAs (miR-335-3p, miR-494-3p, and miR-709) were involved in regulating both myogenic differentiation and muscle fiber type transformation. Our findings may provide insights into the regulatory role of Mdfi in myogenic development and muscle fiber type transformation. The hub miRNAs and genes might provide candidate biomarkers for treating muscle- and metabolic-related diseases. The results warrant our interest in verifying the relationship between Mdfi and hub miRNAs/genes to understand how Mdfi regulates C2C12 cell differentiation and muscle fiber type transformation.

## Data Availability

The datasets presented in this study can be found in online repositories. The names of the repository/repositories and accession number(s) can be found below: NCBI SRA BioProject, accession no: PRJNA725396.

## Glossary

BP: Biological processes
Camk2b: calmodulin-dependent protein kinase II beta
CC: cellular component
Col1a2: collagen type I alpha 2 chain
DEGs: differentially expressed genes
DEMs: differentially expressed miRNAs
Flt1: Fms related receptor tyrosine kinase 1
Gsk3b: glycogen synthase kinase3b
Htr7: hydroxytryptamine receptor 7
Lactb: lactamase beta
Lpar4: lysophosphatidic acid receptor 4
Mdfi-OE: Mdfi overexpression
miRNAs: microRNAs
MF: molecular function
MyHC I: muscle fiber type I
MyHC IIa: muscle fiber type IIa
MyHC IIx: muscle fiber type IIx
MyHC IIb: Muscle fiber type IIb
MYL1: myosin light chain 1
Onecut2: one cut homeobox 2
Pde1b: phosphodiesterase 1B
Pik3cb: phosphatidylinositol-4,5-bisphosphate 3-kinase catalytic subunit beta
Prox1: prospero homeobox 1
Ptger2: prostaglandin E receptor 2
qRT-PCR: quantitative Real-Time PCR
Rnf152: ring finger protein 152
Runx1t1: RUNX1 partner transcriptional co-repressor 1
Ryr3: ryanodine receptor 3
Slc5a3: solute carrier family 5 member 3
Stc1: stanniocalcin 1
Ttn: titin
WT: wild-type
