# Supplementary material for: Putative MicroRNA-mRNA Networks Upon Mdfi Overexpression in C2C12 Cell Differentiation and Muscle Fiber Type Transformation
Source: Front Mol Biosci. 2021 Oct 19;8:675993. doi: 10.3389/fmolb.2021.675993 (PMC8560695; doi:10.3389/fmolb.2021.675993)
Supplement: Supplementary file 2 [file Table1.DOCX]

Table S1. The primer list of differently expressed miRNAs.

| miRNA | Forward primer sequerence (5'-3') |
| --- | --- |
| mmu-miR-190a-5p | TGATATGTTTGATATATTAGGT |
| mmu-miR-335-5p | TCAAGAGCAATAACGAAAAATGT |
| mmu-miR-1258-3p | TTAGGGAATTAGCTCAGCAGTA |
| mmu-miR-344b-3p | CATTTAGCCAAAGCCTGACTGT |
| mmu-miR-10a-5p | TACCCTGTAGATCCGAATTTGTG |
| mmu-miR-504-5p | AGACCCTGGTCTGCACTCTATC |
| mmu-miR-208b-3p | ATAAGACGAACAAAAGGTTTGT |
| mmu-miR-153-3p | TTGCATAGTCACAAAAGTGATC |
| mmu-miR-34b-5p | AGGCAGTGTAATTAGCTGATTGT |
| mmu-miR-133a-5p | GCTGGTAAAATGGAACCAAAT |
| mmu-miR-499-5p | TTAAGACTTGCAGTGATGTTT |
| mmu-miR-6937-5p | TAGCTGTAAGGGCTGGGTCTGTGT |
| mmu-miR-6240 | CCAAAGCATCGCGAAGGCCCACGGCG |
| mmu-miR-494-3p | TGAAACATACACGGGAAACCTC |
| mmu-miR-6236 | GCCGTCGCCGGCAGTCAGG |
| mmu-miR-6239 | TAGCGTTGGATCACTCGGTG |
| U6 | GCGCGTCGTGAAGCGTTC |
