# Supplementary figures and images for: Putative MicroRNA-mRNA Networks Upon Mdfi Overexpression in C2C12 Cell Differentiation and Muscle Fiber Type Transformation
Source: Front Mol Biosci. 2021 Oct 19;8:675993. doi: 10.3389/fmolb.2021.675993 (PMC8560695; doi:10.3389/fmolb.2021.675993)

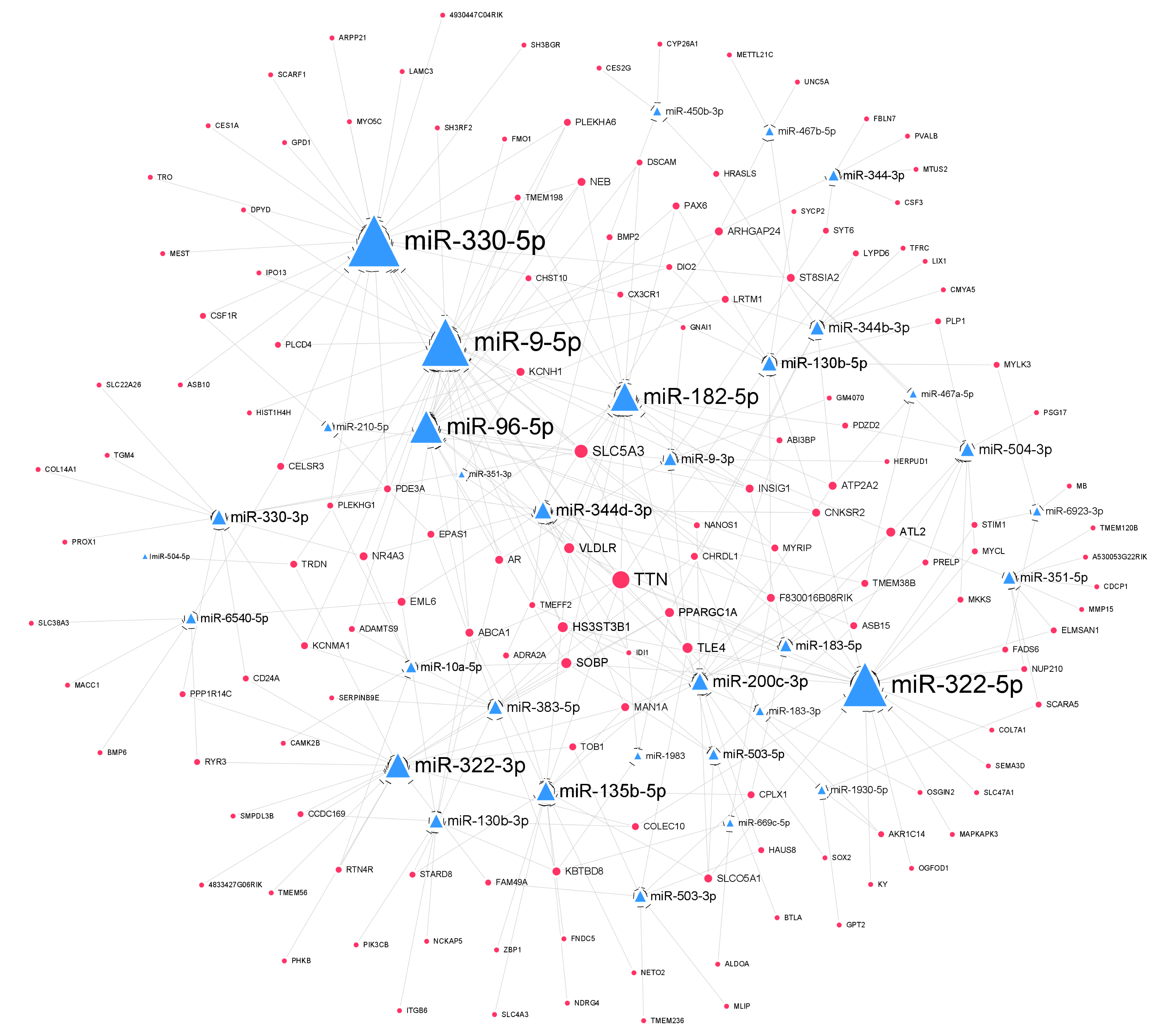

Supplement: Supplementary file 3 [file Image2.TIF]

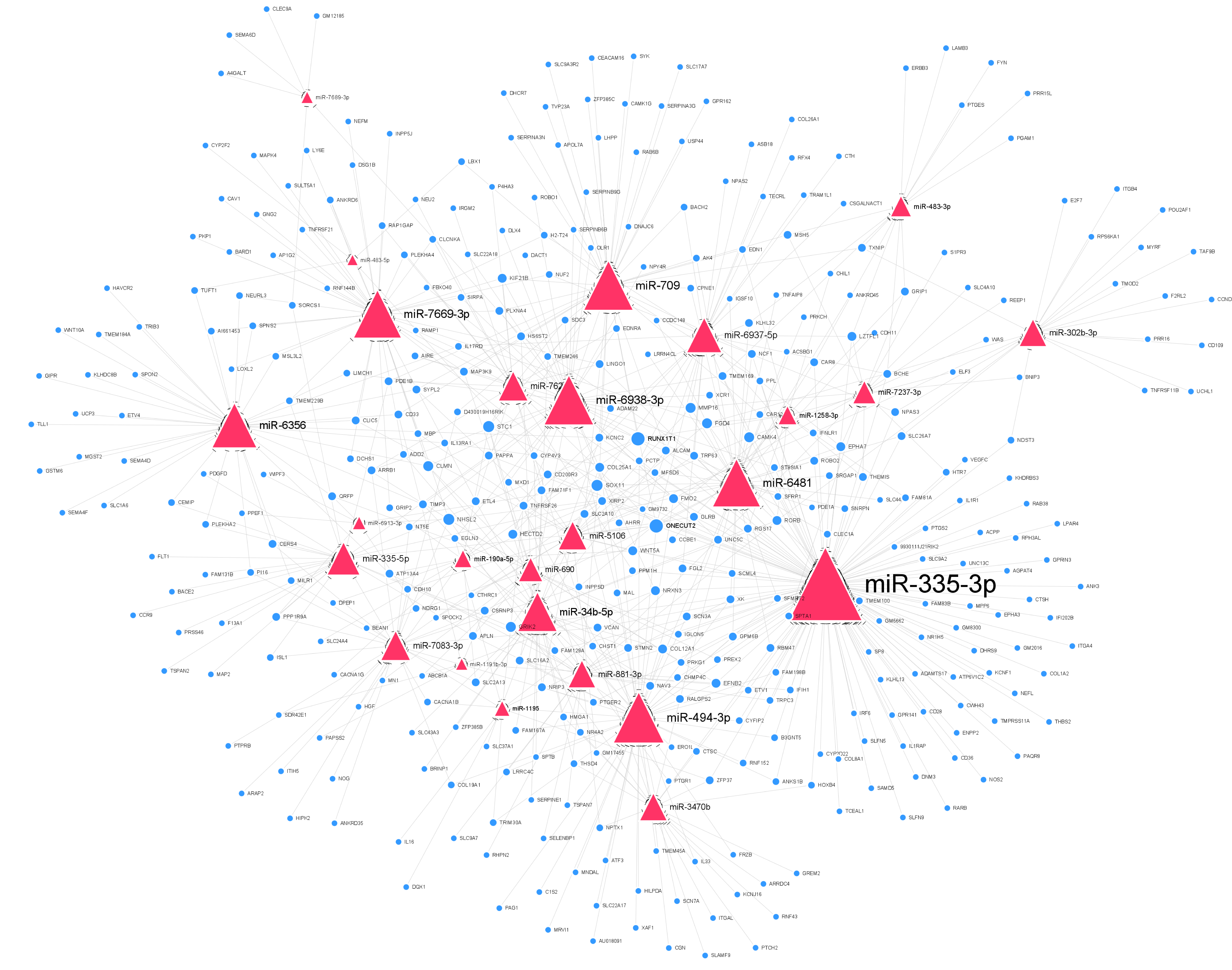

Supplement: Supplementary file 4 [file Image1.TIF]
