# Supplementary material for: Putative MicroRNA-mRNA Networks Upon Mdfi Overexpression in C2C12 Cell Differentiation and Muscle Fiber Type Transformation
Source: Front Mol Biosci. 2021 Oct 19;8:675993. doi: 10.3389/fmolb.2021.675993 (PMC8560695; doi:10.3389/fmolb.2021.675993)
Supplement: Supplementary file 6 [file Table2.DOCX]

**Table S1. Overview of miRNA Sequencing Data.**

| **Samples** | **ID** | **Length** | **Clean reads** | **Q30 (%)** | **GC (%)** | **Mapped Reads (%)** |
| --- | --- | --- | --- | --- | --- | --- |
| Wild-Type 1 | WT1 | 23.68 | 18017760 | 97.97 | 46.67 | 89.62 |
| Wild-Type 2 | WT2 | 23.14 | 8560391 | 97.97 | 46.78 | 89.28 |
| Wild-Type 3 | WT3 | 24.08 | 9660700 | 98.35 | 47.40 | 89.26 |
| Mdfi-Overexpression 1 | Mdfi-OE1 | 22.49 | 6048040 | 97.72 | 48.14 | 90.53 |
| Mdfi-Overexpression 2 | Mdfi-OE2 | 23.21 | 12682374 | 97.92 | 46.37 | 89.51 |
| Mdfi-Overexpression 3 | Mdfi-OE3 | 23.08 | 8525715 | 97.78 | 48.85 | 89.92 |

Samples: Wild-Type 1/2/3 represent normal C2C12 cells. Mdfi-Overexpression 1/2/3 represent overexpression Mdfi in C2C12 cells. ID represents sample serial numbers. Length represents the length of sequencing reads. Clean reads were done after removing impurity reads for raw reads. Q30 % represents the percentage of bases with mass values greater than or equal to 30. GC Content is the G and C base content. Mapped Reads are the percentage of each sample aligned to the mouse reference genome.
